# Multidisciplinary Management of a Fractured Premolar: A Case Report with Followup

**DOI:** 10.1155/2012/192912

**Published:** 2012-07-05

**Authors:** Madhavi R. Kondapuram Seshu, Christopher Leslie Gash

**Affiliations:** ^1^Iscraig Dental Surgery, Conwy, Wales LL32 8LD, UK; ^2^Liverpool University Dental Hospital, Pembroke Place, Liverpool, Merseyside L3 5PS, UK

## Abstract

The general dental practitioner must consider orthodontic extrusion of a tooth when a subgingival defect, such as, crown fracture occurs before prosthetic rehabilitation, especially in the aesthetic zone. Extrusion enables the root portion to be elevated which exposes sound tooth structure for placement of restorative margins. This case report describes the multidisciplinary management of a fractured upper first premolar in a general dental practice. The forced orthodontic eruption is achieved by an endodontic attachment and sectional fixed appliance with an offset placed in the wire. The ability to extrude premolars with this method is complicated by heavy occlusal forces, occlusal interferences, and short clinical crown length. The tooth was restored with a titanium post, composite core, and porcelain fused to metal crown. The entire course of treatment was carried out under National Health Scheme, UK and as a part of vocational training. The 21 months followup showed no change in occlusal contacts or gingival level.

## 1. Introduction

Any subgingival or subosseous extension of a pathologic or traumatic defect that precludes the traditional restorative approach is a possible indication for orthodontic extrusion. Extrusion avoids the loss of a dental unit and simplifies the prosthetic restoration [1]. 

In the normal course of events, bone and gingival movements are produced under low-intensity extrusive forces. When stronger traction forces are exerted, as in rapid extrusion, coronal migration of the tissues supporting the tooth is less pronounced because the rapid movement exceeds their capacity for physiologic adaptation. As well, rapid extrusion must be followed by an extended retention period to allow remodeling and adaptation of the periodontium with the new tooth position, as discussed by Bach et al. in [2]. 

Since Heithersay [3], Ingber [4, 5], and Simon [6] described a method of orthodontically extruding teeth exhibiting transverse fractures in the coronal one-third of the root, numerous authors have suggested additional indications for the procedure. The objectives of forced eruption include preservation of biological width, exposure of sound tooth structure for placement of restorative margins, and maintenance of aesthetics [7]. 

There are several treatment protocols for forced eruption involving removable [7] and fixed appliances depending upon the specific clinical situation. This case demonstrates a technique for orthodontic extrusion of upper premolar with two roots by a sectional fixed appliance and subsequent prosthodontic rehabilitation.

## 2. Case History

Ms SJ, 58-year-old presented with a recent fracture of UL4. She was very keen to avoid dentures. She was a regular attender with good oral hygiene and did not smoke. She presented with a Class II division 2 malocclusion with smile extending to 2nd premolars and attrition in the lower anterior teeth. Her medical history was not contributory.

**Figure 1:**
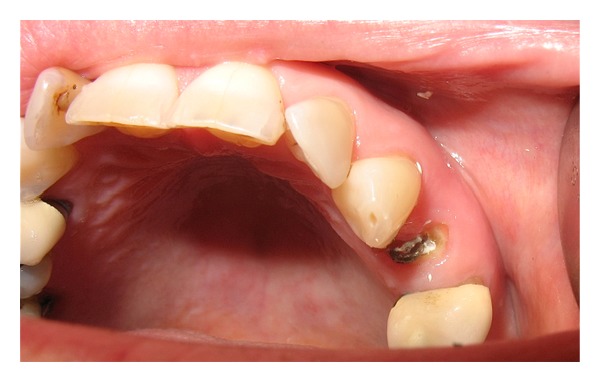
Fractured UL4 & porcelain crown on UL5.

**Figure 2:**
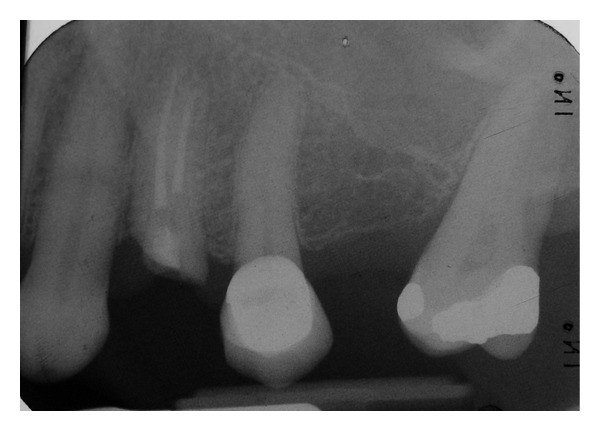
Periapical radiograph of UL4 shows intact root fillings, oblique fracture and a healthy root.

UL4 had been root treated and crowned in the past (>5 years ago). The patient presented with an oblique fracture of UL4 with intact root filling. The fracture line extended 2 mm subgingival on the mesial aspect and was flush with the gingiva on the distal aspect. The gingiva was found to be healthy with normal probing depth. The tooth was asymptomatic, and no periapical pathology was seen. UL3, UL5 were healthy teeth with normal mobility though UL5 has a bonded porcelain crown (Figures 1 and 2).

### 2.1. Plan A

Nonextraction option was chosen because the patient was highly motivated and very keen to save the tooth.

- Orthodontic extrusion (sectional fixed appliance & J hook).
- Titanium post and composite core.
- Porcelain fused to metal crown.

### 2.2. Plan B

Extraction and bridge, if orthodontic extrusion fails. Option of implant was also discussed.

**Figure 3:**
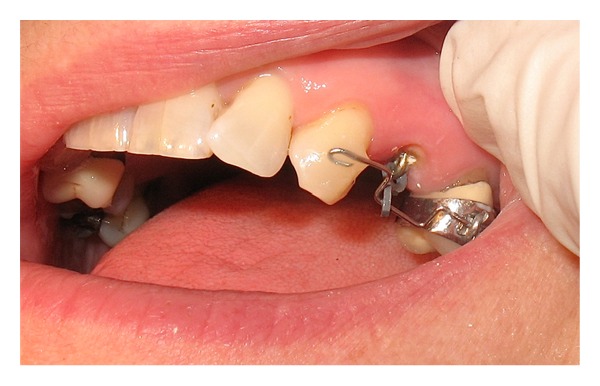
J hook cemented in the root canal is attached by an elastic chain to .018′′ SS sectional wire with an offset for orthodontic traction.

**Figure 4:**
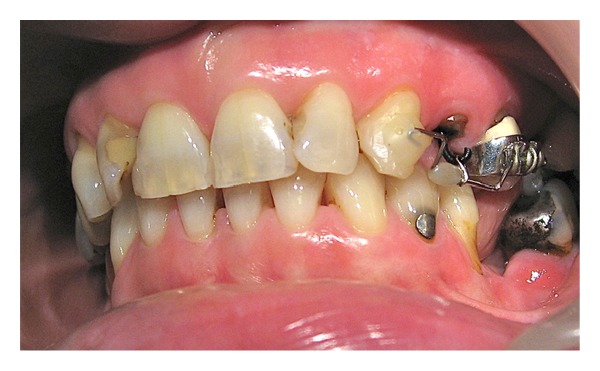
Final stages of extrusion and stabilization by a ligature.

Patient was made aware of the cost (Band C, National Health Scheme), time commitments, and necessary plaque control procedures. Orthodontic extrusion options with fixed and removable appliances were discussed with the patient. Treatment was started after written consent with the sectional fixed appliance as per the patient preference. J hook was fabricated with 1 mm diameter stainless wire and cemented in the root canal with a firm temporary cement (IRM). UL5 was banded as porcelain etch and conditioner were not available in a general dental practice for bonding purposes. A sectional .018′′ SS wire with an occlusal offset was placed between UL3 and UL5. The bend in the sectional wire was made such that the direction of force applied would be along the long axis of the tooth. This was done to prevent labial tipping. The mesial end of the sectional wire was bonded directly to the labial surface of the canine. Orthodontic traction was applied from the J hook by an elastic chain to the sectional .018′′ SS wire (Figure 3). The elastic chain was activated every 10–15 days for 6 weeks. The patient was encouraged to maintain good oral hygiene. The orthodontic extrusion was evident by the visualization of margins of the previously embedded portion of the tooth (Figure 4).

**Figure 5:**
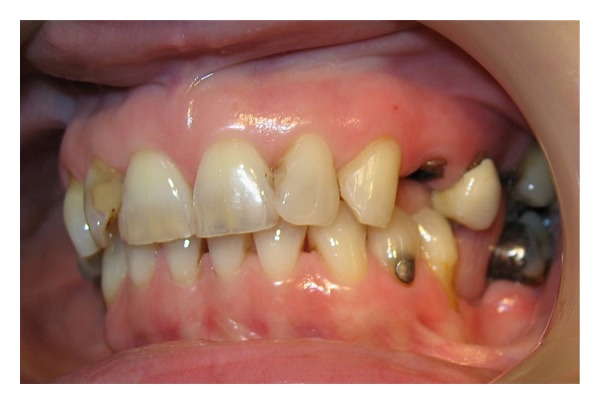
After removal of J hook.

**Figure 6:**
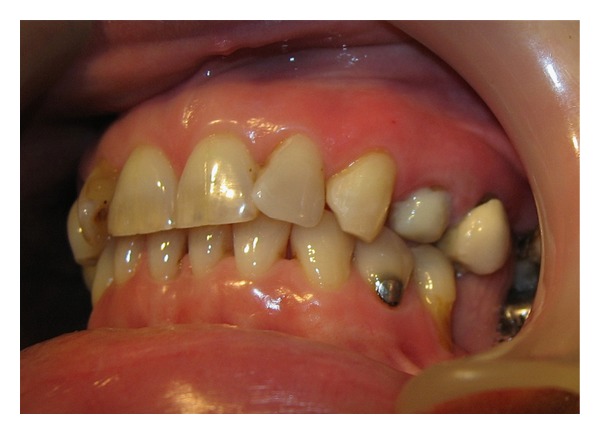
Resin core built on the titanium post before crown preparation.

**Figure 7:**
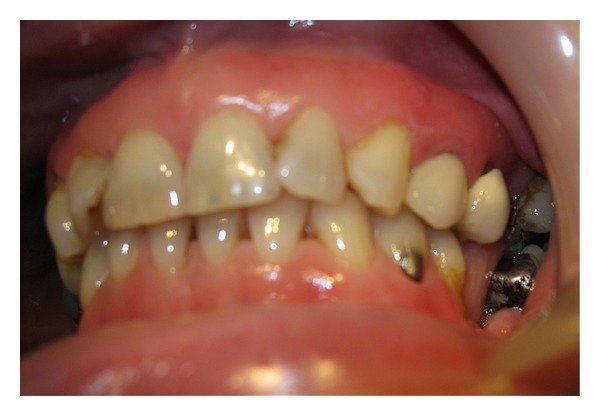
UL4 porcelain crown.

The tooth was stabilized by a ligature from the J hook to the sectional wire for a period of 14 weeks. The J hook was removed (Figure 5), the post space was prepared and a prefabricated titanium post (ParaPost, Coltene/Whaledent) was cemented with Panavia F_2_ [Kuraray America, Inc] a resin-based cement. A heavily filled light cured composite resin core P60 [3M ESPE] was placed and was prepared for a crown (Figure 6). A temporary composite crown was placed for two weeks after which porcelain fused to metal crown was cemented as the final restoration (Figure 7). 

## 3. Followup

21 months later the crown was functioning satisfactorily and had demonstrated no change in occlusal contacts or gingival level relative to the position following cementation. The gingiva was found to be healthy.

## 4. Discussion

This treatment was carried out in a General Dental practice with limited orthodontic materials. Applying this technique to posterior teeth has been suggested but rarely demonstrated in the literature. A study done involving extrusion of more than 100 cases of premolar teeth has been reported by a different technique involving direct bonded brackets and nickel-titanium segmented arch wire [1].

It has been proposed that an important design principle of crown preparation is the provision of a ferrule. This is achieved by “…the parallel walls of dentine extending coronal to the shoulder of the preparation.” [8] It is possible that this extension of dentine, when encircled by a crown, provides a protective effect by reducing stresses within a tooth; the “ferrule effect” [9]. The preparation of a 1 mm ferrule after simulated forced tooth eruption significantly improved the fracture strength of the tooth, and a 2 mm ferrule design was associated with an even higher fracture resistance [10].

Orthodontic extrusion was chosen as the treatment of choice because of aesthetics, good oral hygiene, successful endodontic treatment, and patient motivation. The periodontal health was stable and there was sufficient centric and functional occlusal clearance to allow desired amount of extrusion. The crown root ratio was adequate (at least 1 : 1 after extrusion). 

A study done with magnets reported a force of 50–240 gms for orthodontic extrusion [11]. The force used will vary depending on the physiologic response of the patient and other factors, such as, root surface morphology. The extent of the force exerted can only be approximated, since it is difficult to quantify the force applied. The forces must be adjusted on the basis of the clinically verified speed of extrusion [2]. The force applied was along the long axis of the premolar by a J hook, to prevent tilting of the tooth and an offset was placed in the stainless steel wire.

In most cases, endodontic treatment must be completed first [2]. In this patient, the root of UL4 had been treated successfully in the past and the filling was intact.

Orthodontic extrusion can be carried out by removable or fixed appliances depending on mobility of adjacent teeth, anchorage, and type of force required. Sectional Fixed appliance was chosen due to aesthetics, patient preference, and UL3 and UL5 were deemed suitable for anchorage.

Various extrusion methods are available depending on the clinical situation with a variety of mechanical strategies to control the forces applied. Forces to the tooth can be applied through a bracket, a rigid stainless wire in the root canal or a temporary clinical crown cemented on a post. Traction to the wire can be applied by an elastic chain, a looped wire, or a spring as discussed by Bach et al. in [2]. J hook was chosen as the tooth material was insufficient to bond a bracket and is easy to fabricate. Traction was initially provided with an elastic chain and completed with a ligature. Other innovative methods of forced extrusion include magnets [11], “Forced extruder” [13].

Practically there is always some movement of the gingiva and alveolar bone with the root but considerably less than if the extrusion was completed with lesser forces at a slower rate [14]. This coronal migration of tissues could mask some of the extrusion achieved. Some authors recommend a single fiberotomy procedure when the movement is complete [15]. In-depth studies on human subjects to demonstrate the usefulness of this procedure and to define the frequency have yet to be carried out.

The time required for forced eruption varies with the clinical situation. After active movement the tooth should be stabilized for reorganisation of PDL fibres and bone remodelling and to prevent relapse. In general, 3–6 weeks of stabilization should be sufficient [16] but some studies indicate that a stabilization period of 7–14 weeks is required [3, 5]. In this case 6 weeks of active extrusion was followed by 14 weeks of stabilization.

If less than one-half of the coronal tooth structure is remaining on a pulpless tooth, it is usually advisable to place a post and core; thereby providing adequate connection of the root structure to the coronal core [17]. Titanium post was used because of ease of use and availability. Composite resin P60 (3M ESPE) was used as the core material due to its strength, ease of use, and radiopacity.

The treatment described requires time, commitment, and motivation from the patient and the dentist. However, it is less destructive of tissue than the other treatment options available and is more natural to a patient than a denture. It can be a useful tool in the armamentarium of a general dentist.
